# Autophagy provides a conceptual therapeutic framework for bone metastasis from prostate cancer

**DOI:** 10.1038/s41419-021-04181-x

**Published:** 2021-10-05

**Authors:** YouZhi Wang, Ning Wu, Ning Jiang

**Affiliations:** 1 Department of Urology, Tianjin Institute of Urology, The Second Hospital of Tianjin Medical University, 300211 Tianjin, China; 2 The First Department of Breast Cancer, Tianjin Medical University Cancer Institute and Hospital, National Clinical Research Center for Cancer, Key Laboratory of Cancer Prevention and Therapy, 300060 Tianjin, China; 3 Tianjin’s Clinical Research Center for Cancer, Key Laboratory of Breast Cancer Prevention and Therapy, Tianjin Medical University, Ministry of Education, 300060 Tianjin, China

**Keywords:** Cancer models, Prostate cancer

## Abstract

Prostate cancer is a common malignant tumor, which can spread to multiple organs in the body. Metastatic disease is the dominant reason of death for patients with prostate cancer. Prostate cancer usually transfers to bone. Bone metastases are related to pathologic fracture, pain, and reduced survival. There are many known targets for prostate cancer treatment, including androgen receptor (AR) axis, but drug resistance and metastasis eventually develop in advanced disease, suggesting the necessity to better understand the resistance mechanisms and consider multi-target medical treatment. Because of the limitations of approved treatments, further research into other potential targets is necessary. Metastasis is an important marker of cancer development, involving numerous factors, such as AKT, EMT, ECM, tumor angiogenesis, the development of inflammatory tumor microenvironment, and defect in programmed cell death. In tumor metastasis, programmed cell death (autophagy, apoptosis, and necroptosis) plays a key role. Malignant cancer cells have to overcome the different forms of cell death to transfer. The article sums up the recent studies on the mechanism of bone metastasis involving key regulatory factors such as macrophages and AKT and further discusses as to how regulating autophagy is crucial in relieving prostate cancer bone metastasis.

## Facts

- The most usual metastatic location in prostate cancer is bone metastasis.
- Metastatic prostate cancer has a high death rate in advanced prostate cancer patients, but there is no good treatment model so far.
- There are many studies on the association between autophagy and cancer metastasis; however, it is rarely correlated to bone metastases models of prostate cancer.

## Open questions

- Can we inhibit autophagy activity of cancer cells to inhibit bone metastases from prostate cancer by linking classical signal transduction pathways?
- Can this autophagy model of bone metastasis be established successfully? Does it inhibit tumor metastasis in the primary site or inhibit tumor growth that has already been metastatic?
- To what extent would deeper understanding of autophagy and bone metastasis help improve human survival?

## Introduction

Prostate cancer (PC) is the second most common malignancy in males [1]. With improvement in diagnosis and treatment, reports of cases with PC have been increasing every year in China [2]. The prostate is made up of two layers of epithelial cells: basal epithelial cells and luminal epithelial cells [3]. Interestingly, these two cell types express profile of androgen receptors (ARs) differently. Nearly all luminal epithelial cells express AR and AR signal is necessary for them to survive; however, basal epithelial cells are usually AR-negative and hence insensitive to castration. Charles Huggins reported that PC relies on AR signal, similar to luminal epithelial cells, based on which human PC has been widely deemed to be derived from luminal epithelial cells. Like many other types of cancer, PC cells exhibit heterogeneity generally [4–6]. Metastatic PC accounts for only 5–6% of new cases of PC in the European and American populations, while in China, the rate is as high as 54% [7, 8]. Of note, among all PC metastases, bone metastases are predominant [9].

Autophagy is a gene-regulated process in eukaryotes. It regulates organelles and protein degradation so that they can be recycled [10, 11]. In normal cells, autophagy can inhibit the occurrence of tumors and prevent them from becoming cancerous. Contrarily, autophagy drives the migration and proliferation of tumor cells [12, 13]. Merging lysosomes with autophagosomes can enhance the sensitivity of tumor cells to various treatments, including DNA damage agents, anti-hormone therapy, and radiotherapy. Autophagy acts mainly by regulating the phosphoinositide-3 kinase (PI3K)/AKT pathway [14, 15].

At present, the studies of bone metastasis from PC are not extensive enough, and most of the studies have not been combined with autophagy. However, autophagy plays an indispensable role in tumor metastasis. So it is important to provide a model of bone metastasis and a conceptual treatment model for PC. This paper introduces the relationship between tumor and autophagy and reviews all the studies affecting bone metastases from PC in the past decade, as well as the relationship between these mechanisms and autophagy. Moreover, we discussed whether autophagy is important in the process of bone metastasis from PC, and how to control it to inhibit tumor growth.

## Metastasis of PC

The major reason of PC patients’ death is metastatic disease [16]. The first site of metastasis are usually the lymph nodes that are near the main tumors, followed by liver, lung, and bone [17]. Bone metastases from PC most commonly present as osteoblastopathy with osteolytic function that can cause frequent fractures, hypercalcemia, and sharp pain [18, 19]. Epithelial–mesenchymal transition (EMT) plays a key role in the metastases of different types of cancers, such as PC. Although its role in body is controversial, it has been extensively reviewed elsewhere. PCs pass through EMT, spread to circulating tumor cells, conquer obstacles of body and establish bone metastasis, cross the sinus wall and bone marrow stroma, and move to the endosteum appearance through the sinus vessels in the bone marrow cavity [20–22].

**Fig. 1:**
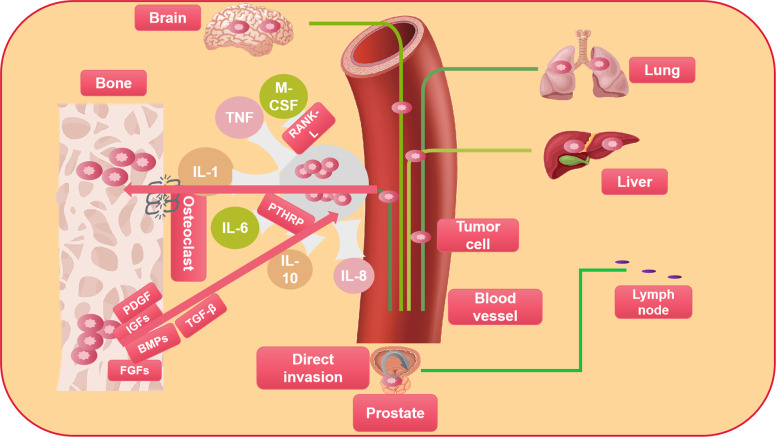
The basic metastatic sites and pathways of PC and the vicious circle of osteolytic metastasis. Prostate cancer mainly metastases to the bone, but some tumor cells also spread through the blood to the viscera, such as the lung, liver, and brain. In addition, PC can also develop direct spread and lymph node metastasis. Parathyroid hormone-related peptides secreted by tumor cells are the major stimulators of osteoclast formation. Additionally, cancer cells also generate other factors, the formation of which promote osteoclasts, including prostaglandin E2 (PGE2), interleukin, macrophage colony-stimulating factor (M-CSF), and tumor necrosis factor. These factors increase the expression of nuclear factor-KB ligand receptor activators (RANKL), which acts on preosteoclast to induce bone resorption and osteoclast formation directly. Bone resorption releases factors including platelet-derived growth factor (PDGF), fibroblast growth factors (FGFs), insulin-like growth factors (IGFs), bone morphogenetic proteins (BMPs), and transforming growth factor b (TGF-b), which can enhance the parathyroid hormone–related peptide production through cancer and growth factors, thereby promoting tumor growth. Tumor growth and bone destruction are further increased because of the symbiotic connection between tumor growth and bone destruction.

When PC cells localize the bone marrow, the mutual effect between the cancer cells and the microenvironment of bone leads to a bad circle of the destruction and regeneration of bone, a process which supports the survival of cancer cells and the growth of tumors [23–25]. PC cells secrete growth factors, including adrenomedullin, endothelin 1, bone morphogenetic proteins, fibroblast growth factors, and platelet-derived growth factor (PDGF), which can stimulate osteoblasts to activate and form new bone by paracrine signals [26]. In addition, protease secreted by tumor (urokinase-type plasminogen activator, prostate-specific antigen (PSA), and matrix metalloproteinases) promotes the release of osteoblast-induced growth factors, such as insulin-like growth factors, PDGF, and transforming growth factor β (TGF-β), which further enhances the differentiation of mesenchymal stem cells into osteoblasts [27]. Activated osteoblasts result in increased receptor activator of nuclear factor kappa-Β ligand (RANKL) concentration, hypocalcemia, and parathyroid hormone release that respond to hypocalcemia. Both activate osteoclasts and subsequently release factors such as TGF-β through osteoclast-mediated bone resorption [28]. The factors increase the survival ability of PC cells, produce proteins including parathyroid hormone associated proteins, and promote the production of RANKL and osteoprotectin downregulation in osteoblasts and stromal cells. This results in further osteoclast activation. Wnt signal transduction pathways activated in PC cells is also important in driving osteogenic differentiation. Prostate transmembrane protein androgen-induced 1 is a TGF-β1-induced gene that inhibits bone metastases of PC by blocking TGF-β signal transduction by interacting with HECT E3 ubiquitin ligase and Smad2/3 [29]. Monoamine oxidase A, a mitochondrial membrane-conjugated enzyme that can catalyze degradation in biology and diet, has shown its crucial role in the EMT process through oxidative deamination, promoting bone metastasis and inducing RANKL expression and interleukin-6 (IL-6) by activating paracrine Shh signal transduction in osteoblasts [30, 31]. To sum up, the continuous growth of metastatic PC cells involves a dynamic bone remodeling process that results from the interaction of cancer cells, osteoblasts, and osteoclasts (Fig. 1).

## Different factors affecting bone metastasis from PC

**Fig. 2:**
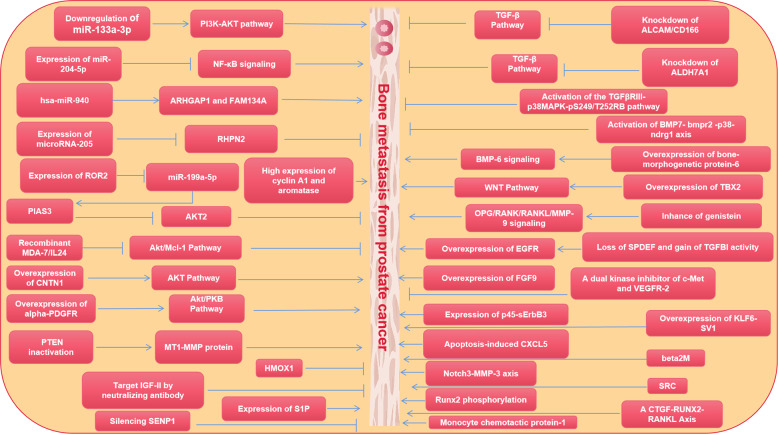
Different factors that inhibit or promote bone metastasis from PC. Influence of different signaling pathways and drugs on bone metastasis from PC.

To date, many studies have been published on pathways that influence bone metastasis from PC. Many articles have made in-depth discussions on bone metastasis, some of which are based on animal model verification, some of which focus on case collection and in-depth immunohistochemistry, and more articles focus on more detailed studies on the cellular mechanism. In order to better ensure the accuracy and timeliness of the mechanism, we selected all the articles (2000–2021) that affect bone metastasis from PC. All the mechanisms affecting bone metastasis from PC can be separated into the following types: (1) PI3K/Akt signal transduction pathway is dominant [32–36]; (2) TGF-β signal transduction pathway was dominant [37–39]; (3) miR-RNA was dominant [34, 35, 40–42]; (4) other signal transduction pathways and drugs [36, 39, 43–64] (Fig. 2).

## Autophagy and PI3K/AKT/mammalian target of rapamycin (mTOR)

Autophagy is a very conservative cellular metabolic process that can be activated under different cellular pressure environments [65]. Eukaryotic cells transport unnecessary and possibly harmful cytoplasmic materials to lysosomes for digestion [66]. At this point, the major pathways of autophagy substrate transfer to lysosomes are macrophages, chaperon-mediated autophagy, and microautophagy [67].

Autophagy responds to a variety of cytotoxic injuries, thereby enhancing survival and preventing damage to cells lacking energy or nutrients. Therefore, adapting to the changing circumstances, autophagy needs rigorous regulation so that it can respond to different stimuli accurately [68, 69]. Autophagy is maladjusted in many conditions, even in tumors. Autophagy regulation is expected to be a cancer therapy strategy [70, 71]. BECN1 is a center protein forming the BECN1–VPS34–VPS15 complex by aggregating cofactors, which triggers the cascade of autophagy protein [72]. AMBRA1 is important in autophagy regulation. Some studies have demonstrated that VPS34, BECN1, and AMBRA1 are parts of autophagy promoter complex. Besides, AMBRA1 downregulation shortens the ability of BECN1 and the activity of kinase VPS34 to connect with its VPS34 connection [73].

The autophagy main mechanism is composed of about 40 proteins in yeast. And autophagy is mainly caused by unc-51 like autophagy activating kinase 1 (ULK1) complex [10, 74]. ULK1 complex in human is composed of the FIP200 (FAK family kinase interacting protein of 200 kDa; also called RB1CC1) scaffold, the ULK1 protein kinase and the Hop/Rev7/Mad2 domain, including ATG101 protein and ATG13 protein [74]. Moreover, ULK1 is regulated by energy status and amino acid by mTORC1 (mechanistic target of rapamycin-1) and AMPK (AMP activated protein kinase) kinases. When mTORC1 is activated, ATG13 and ULK1 phosphorylation inhibit autophagy, thereby reducing ULK1 kinase activity [11]. Besides, the Ras-like G protein RalB can trigger the catalytic activity of BECN1-VPS34 and ULK1 complexes to promote biogenesis of autophagosome by directly binding to Exo84 [75, 76]. Another research discovered that ULK1 activation on mTOR inhibited S14 and promoted the activity of the VPS34 complex containing ATG14L [77].

Cancer cell autophagy involves many pathways. For example, the signal transduction pathway of JUK/cJun, AMPK/mTOR/p70S6K, Hh, ERK/AKT/mTOR/STAT3/Notch, Wnt/beta-catenin, Notch1/Hes-1, epidermal growth factor receptor (EGFR)/Ras/MEK/ERK, P38MAPK, and EGFR/Ras/MEK/ERK [77–79]. In particular, the PI3K/AKT/mTOR pathway as a regulatory pathway for autophagy has attracted extensive attention [14, 80]. The mTOR is the central inspection point for autophagy negative regulation and antitumor agents through inhibiting the PI3K/AKT/mTOR pathway to stimulate autophagy [81]. At present, autophagy-related inhibitors or promoters have been successively discovered and proved. To better verify the related role of Akt and autophagy, different compounds and gene methods were screened to affect the PI3K/AKT/mTOR signal transduction pathway by regulating autophagy (Table 1).

**Table 1:** Compounds and genes known to regulate autophagy and that are associated with the PI3K/AKT/mTOR signaling pathway.

Compounds and genetic methods	Effect	Reference
3-methyladenine	A PtdIns3K inhibitor that effectively blocks an early stage of autophagy by inhibiting the class III PtdIns3K but not a specific autophagy inhibitor. 3-MA also inhibits the class I PI3K and can thus, at suboptimal concentrations in long-term experiments, promote autophagy in some systems, as well as affect cell survival through AKT and other kinases. 3-MA does not inhibit BECN1-independent autophagy	[93–95]
10-NCP	10-(40-*N*-diethylamino)butyl)-2-chlorophenoxazine; an AKT inhibitor that induces autophagy in neurons	[96]
Akti-1/2	An allosteric inhibitor of AKT1 and AKT2 that promotes autophagy in B cell lymphoma	[97]
ESC8	A cationic estradiol derivative that induces autophagy and apoptosis simultaneously by downregulating the MTOR kinase pathway in breast cancer cells	[98]
Everolimus	An inhibitor of MTORC1 that induces both autophagy and apoptosis in B cell lymphoma primary cultures	[99]
KU-0063794	An MTOR inhibitor that binds the catalytic site and activates autophagy	[100, 101]
NVP-BEZ235	A dual inhibitor of PIK3CA/p110 and the MTOR catalytic site that activates autophagy	[102–104]
Wortmannin	An inhibitor of PI3K and PtdIns3K that blocks autophagy, but not a specifific inhibitor	[105, 106]

Supplementary: this table is not meant to be complete, as there are many compounds and genetic methods that regulate autophagy, and new ones are being discovered routinely.

## Autophagy and bone metastasis: foe or friend?

**Fig. 3:**
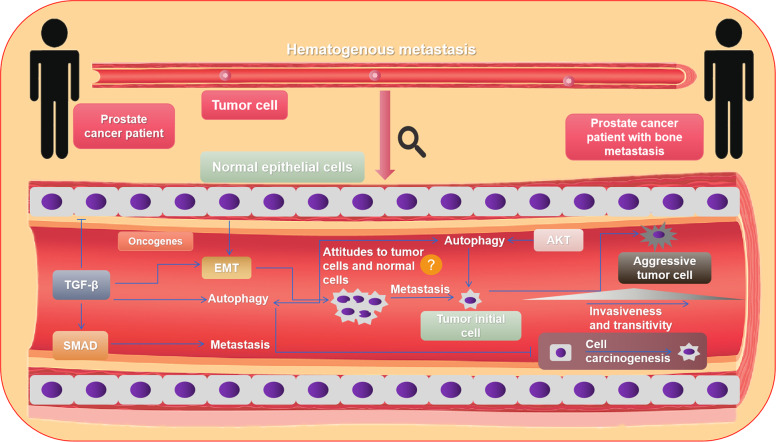
The mechanism of metastasis via blood in advanced bone metastasis from PC. Bone metastasis from PC mainly occurs via blood, where multiple signaling pathways are involved. Focus on the AKT signaling pathway, which has a remarkable relationship with autophagy, and TGF-β signaling pathway, which can induce EMT formation and release Smad protein to promote tumor metastasis.

The progress of autophagy, Akt pathway, and tumor bone metastasis in other tumors is also limited, and the research is mainly focused on osteosarcoma [82, 83]. Further results show that autophagy is involved in the in vivo and in vitro model of osteosarcoma development and metastases [83]. Spreading metastatic cells must face a number of adverse factors, such as separation from extracellular matrix, immune cell attack, and an environment devoid of oxygen or growth factors. These conditions lead to increased production of cellular ROS, insufficient energy status, and DNA damage [84, 85]. Apoptosis is stimulated by low level of death signals, while high levels often cause necroptosis. Because of the function of the necroptosis and apoptosis mechanisms, cells with metastasis capability from major tumors cannot metastasize in a large size successfully [71, 86]. Autophagy is quite variable compared to apoptosis and necrosis. On the one hand, autophagy improves the large adaptation of metastatic cells under stress and further to combat necrosis and apoptosis. In addition, autophagy can reduce metastasis by inhibiting blocking inflammatory immune cell infiltration and tumor necrosis [71, 87]. Interestingly, excessive autophagy induces metastasis cell death [88, 89]. Autophagy is currently being investigated as both a modulator and target in PC. While it can have very subtle effects on cells, it is quite different from the internal environment of the cell; specifically, autophagy interacts with cancer cells (Fig. 3). Autophagy inhibits the carcinogenesis of normal cells and promotes the growth of tumor cells [71]. Moreover, cell growth or death simultaneously affects the autophagic activity in the tumor environment [90, 91]. Therefore, to learn the relationship between metastatic tumor and autophagy, it is necessary to learn the relationship between PC cells and autophagy.

Most animal models of preclinical PC metastasis have been made by directly injecting well-defined cell lines into recipients with immune deficiency. Immunodeficient animals with immune deficiency are often necessary for cell line injection model, especially where the cell type of origin is different from the host [44]. The animal model is difficult to achieve success, as the cell line, mouse strain, and injection site quantified as tumor extraction rates need to be assured (Table 2). Various cell lines have been established (ATCC sold 14 prostate tissue cell lines). The width of useful cell lines is noteworthy because every cell line has their own genetic spectrum that can be applied for modeling various aspects of PC, such as reactivity of hormone, antigenicity, and ability of metastasis. Established PC cell lines are most commonly derived from DU145, PC3, or LNCaP cells, though other cell lines exist. Primary cell lines are usually defined based on some biochemical features, such as PSA expression or AR dependency, and can be selected as needed for experimental or therapeutic evaluation (Table 3). Similarly, a wide variety of transfer cell lines can be used in animal models to study metastasis.

**Table 2:** Commonly used cell lines in the translational models of prostate cancer.

Prostate cancer cell lines	Source of cell	Type of bone metastasis	Androgen receptor (AR)+	Prostate-specific antigen (PSA)+	Reference
PC3	Bone	Osteolytic	−	−	[107–110]
LNCaP	Supraclavicular lymph node	Osteoblastic	+	+	[107, 109, 110]
LNCaP-C4-2B	Subline of LNCaP	Osteoblastic	−	+	[107, 108, 111–115]
DU145	Brain	Osteolytic	−	−	[107, 109, 116–118]

**Table 3:** Translational models of prostate cancer bone metastasis.

Cell lines	Inoculation method	Number of cells used	Reference
PC3	Orthotopic	200,000 or 1 mm^3^ of a subcutaneous tumour	[119, 120]
LNCaP	Orthotopic	1,000,000	[121]
LNCaP C4-2B	Subcutaneous	200,000	[120]
LNCaP C4-2	Orthotopic	1,000,000	[111]
	Subcutaneous	1,000,000	[111]

After autophagy stimulation, LC3 puncta, a typical autophagy marker, were not detected in DU145 cells, indicating a lack of autophagy function in this cell line [92]. And published researches have revealed that the autophagic activity of PC3/LNCaP cells is higher than that of other PC cells [92]. Therefore, it is appropriate to select these two cell lines to construct the model. By adding autophagy inhibitors that are associated with AKT pathway, we can inhibit cells’ autophagy activity further, and the size and invasiveness of bone metastatic tumor could be significantly reduced.

## Conclusion and future direction

In this article, we primarily considered whether metastatic bone tumors from PC could be inhibited or induced by altering the autophagic activity of PC cells. First, by summarizing the published studies on the pathways affecting bone metastasis from PC, we found that the AKT signaling pathway is important in bone metastasis from PC. Then the relationship across AKT, EMT, autophagy, and metastatic tumor was gradually elucidated through careful logical scrutiny, so as to judge the feasibility of this treatment scheme. Finally, we concluded that there is a strong correlation between AKT-related signaling pathway and autophagy, and it can affect the formation of bone metastasis of PC by influencing the internal connection between them. Moreover, the influence of autophagy on the occurrence and development of bone metastasis was explored. Inhibiting the autophagic activity of tumor cells by considering to add autophagy inhibitors related to the AKT signal transduction pathway enabled further exploration of the treatment methods to inhibit bone metastasis. This design could provide a macroscopic direction for exploring the diagnosis and therapy of autophagy and bone metastasis from PC.

## Data Availability

The datasets used and analyzed during the current study are available from the corresponding author on reasonable request.
