# Correction: Autophagy provides a conceptual therapeutic framework for bone metastasis from prostate cancer

**DOI:** 10.1038/s41419-021-04341-z

**Published:** 2021-10-29

**Authors:** YouZhi Wang, Ning Wu, Ning Jiang

**Affiliations:** 1 Department of Urology, Tianjin Institute of Urology, The Second Hospital of Tianjin Medical University, 300211 Tianjin, China; 2 The First Department of Breast Cancer, Tianjin Medical University Cancer Institute and Hospital, National Clinical Research Center for Cancer, Key Laboratory of Cancer Prevention and Therapy, 300060 Tianjin, China; 3 Tianjin’s Clinical Research Center for Cancer, Key Laboratory of Breast Cancer Prevention and Therapy, Tianjin Medical University, Ministry of Education, 300060 Tianjin, China

**Keywords:** Cancer models, Prostate cancer

Correction to: *Cell Death and Disease* 10.1038/s41419-021-04181-x, published online 05 October 2021

The original version of this article unfortunately contained a mistake in the Funding section. The correction in funding is: This work was supported by National Natural Science Foundation of China [grant numbers 81872079 and 81572538], the Natural Science Foundation of Tianjin City [grant numbers 11JCZDJC19700, 16JCZDJC34400, 20140117, and 2010KZ95] and Tianjin Municipal Bureau of Public Health [grant numbers ZC20131]. The original article has been corrected.

